# Analysis of lncRNAs and mRNA Expression in the ZBTB1 Knockout Monoclonal EL4 Cell Line and Combined Analysis With miRNAs and circRNAs

**DOI:** 10.3389/fcimb.2021.806290

**Published:** 2021-12-09

**Authors:** Junhong Wang, Xiaoxu Li, Yiyuan Lu, Quntao Huang, Yu Sun, Mingyang Cheng, Fengdi Li, Chunwei Shi, Yan Zeng, Chunfeng Wang, Xin Cao

**Affiliations:** ^1^ College of Veterinary Medicine, Jilin Agricultural University, Changchun, China; ^2^ Jilin Provincial Engineering Research Center of Animal Probiotics, Jilin Agricultural University, Changchun, China; ^3^ Key Laboratory of Animal Production and Product Quality Safety of Ministry of Education, Jilin Agricultural University, Changchun, China

**Keywords:** Zbtb1, EL4, lncRNA, mRNA, RNA-seq

## Abstract

In previous experiments, we identified the effect of deletion of the Zbtb1 gene on circRNAs and microRNAs. In this study, we examined the expression profiles of lncRNAs and mRNAs using the RNA-seq method for Zbtb1-deficient EL4 cells and performed a clustering analysis of differentially expressed lncRNAs and mRNAs. GO term histograms and KEGG scatter plots were drawn. For the experimental results, a joint analysis was performed, which predicted the regulatory relationships among lncRNAs, mRNAs, microRNAs and circRNAs. For the regulatory relationship between lncRNAs and target genes, the chromatin structure and the degree of openness were verified for the possible target gene locations regulated by lncRNA using experimental methods such as Hi-C and ATAC-seq. Ultimately, the possible differential regulation of the Brcal and Dennd5d genes by lncRNAs and the differential changes in transcription factor binding sites in the promoter region were identified. For neRNA-regulated target genes with significantly differentially expressed mRNAs, a combined screen was performed, and the final obtained candidate target genes were subjected to GO and KEGG term enrichment analyses. Our results illustrate that the Zbtb1 gene can not only function as a regulatory factor but also regulate EL4 cells from multiple perspectives based on ceRNA theory.

## Introduction

The Zbtb1 family consists of a different set of transcription factors, and Zbtb1 plays an important role in lymphocyte development (Zhu et al., 2018). The Zbtb protein family also plays an important role in cancer, such as in the relationship between Zbtb7 and endometrial cancer cells and bladder cancer cells (Guo et al., 2017; Geng et al., 2020).

RNA is a single strand formed by transcription of a single DNA strand using the principle of base complementary pairing. RNA is classified as mRNA, miRNA, tRNA, rRNA, ncRNA, etc. Long noncoding RNAs (lncRNAs) are a heterogeneous class of noncoding RNAs that play an important role in gene regulation and are associated with a variety of genetic diseases and cancers (Dangelmaier and Lal, 2020; Ramakrishnaiah et al., 2020). LncRNAs play important functions in many fields, mainly divided into epigenetic regulation, transcriptional regulation and posttranscriptional regulation, can transcriptionally regulate mRNA expression in cis or in trans, and can also bind to microRNAs as ceRNAs to regulate mRNA expression (Yang et al., 2021). Over the past decade, there has been growing evidence that the genomes of many species are commonly transcribed, resulting in numerous LNcRNAs. At the same time, it is now recognized that many types of DNA regulatory elements, such as enhancers and promoters, regularly initiate bidirectional transcription (Serebreni and Stark, 2021). Therefore, the discovery of functional noncoding transcripts from a large transcriptome is a top priority and challenge for the LNCRNA field (Kopp and Mendell, 2018).

The transcriptome refers to the collection of all transcription products in a given cell in a given state, including mRNA and noncoding RNA; research on noncoding RNA has focused on small RNA, lncRNA and circRNA, which have regulatory roles (Luo et al., 2020). Whole-transcriptome sequencing study based on second-generation sequencing technology can analyze mRNA, lncRNA, circRNA, small RNA in the same sample at the same time and explore the transcriptional regulation behind life phenomena in greater depth (Ning et al., 2021). By studying the interaction of coding and noncoding RNAs, scientific questions can be addressed more comprehensively, and the findings can be refined.

## Materials and Methods

### Sample Preparation

EL4 Cell Line With Zbtb1 Gene Knockout were constructed and cultured in a previous experiment (Wang et al., 2021).

### Experimental Procedure

The experimental procedure includes RNA extraction, RNA sample quality testing, library construction, library purification, library detection, library quantification, generation of sequencing clusters, and up-sequencing.

### Analytical Process

Transcriptome sequencing is based on the Illumina HiSeq sequencing platform to study all mRNAs transcribed from a specific tissue or cell at a given time and is the sum of all RNAs that can be transcribed from a specific cell in a given functional state, including coding mRNAs and noncoding RNAs.

### Data Filtering and Transcript Assembly Analysis

During sequencing, low-quality data need to be filtered to remove contaminations and splice sequences. The reads sequenced were assembled using the assembly software StringTie (v1.0.4), and then the corresponding assembly results for each sample were combined as the final transcript results. Based on the gene structure annotation files, the transcript sequences of each sample were assembled using StringTie software. During the assembly process, paired reads of the reference gene region were assembled preferentially, while new transcripts were constructed for the reads that did not match in pairs. For the assembly results, we used the corresponding statistical software for statistical analysis, and then, based on the existing gene structure annotation reference files of the species, we used Cuffmerge software to merge, deduplicate and optimize the transcript structure information obtained from the assembly of each sample to obtain the final reference transcript structure file. Meanwhile, the sequence information corresponding to these transcripts was extracted using gffread software and the reference genome information of the species.

### LncRNA Identification and Prediction

(1) For other kinds of RNA data, transcripts that overlap with known protein-coding, miRNA, tRNA, snoRNA, rRNA and pseudogene annotation regions in the transcriptome were filtered out using the latest genome annotation information provided by UCSC, Ensembl and GENCODE. (2) Filtering for lncRNA features was based on the GENCODE v7 database statistics of available lncRNA features, filtering transcripts containing only one exon, filtering transcripts below 200 bp in length, and filtering transcripts with less than 3 reads. (3) Transcripts containing protein structural domains were filtered. (4) Transcripts with protein-coding potential were filtered.

### LncRNA Statistics

For the annotation of known lncRNAs, lncRNA information from authoritative databases such as the Ensembl, Gencode, and UCSC databases were first integrated for annotation. Similarly, we used Cuffcompare software for annotation. The lengths of the obtained lncRNAs and the numbers of exons contained were counted using the program, and the information on the position of lncRNAs on the reference genome was also used to classify the obtained lncRNAs into the following three categories: intergenic lncRNA, intronic lncRNA, and antisense lncRNA. The number of individuals was also counted.

### Prediction of LncRNA Target Genes

Cis-regulation is usually a mode of action in which DNA sequences on the same chromosome directly regulate the expression of other neighboring genes. We used the genomic annotation information of the predicted obtained lncRNAs and the species, with reference to genomic information, for the identification of possible cis-acting target genes of lncRNAs. Generally, lncRNAs that are transcribed in the promoter region of a gene in the same direction as the target gene usually promote expression to the target gene and transcription in the reverse direction as repression of target gene expression. In determining the promoter region of a gene, we selected a region within 3000 bp upstream of the gene to act as the promoter region of that gene. The Bedtools intersect method was used for this analysis. Transregulatory action is another method by which lncRNAs regulate gene actions, in which lncRNAs and target genes recognize each other directly by base pairing without considering positional relationships. Here, we used Blast (V2.3.0+) software to identify the possible trans target genes of lncRNAs.

### Gene Expression Analysis

A direct reflection of a gene’s expression level is the gene’s abundance; the higher the degree of gene abundance, the higher the level of gene expression. Both lncRNA and mRNA gene expression calculations were performed using Rsem software (V1.2.6), which uses the FPKM (fragments per kilobase per million reads) (Marioni et al., 2008) method to calculate gene expression. The input data for differential gene expression are the read count data obtained from the gene expression level analysis. For samples with biological replicates, gene differential analysis was performed using DESeq2 (V1.6.3) in the Bioconductor software package.

### Clustering Analysis of Differentially Expressed Genes

Clustering analysis is a hierarchical clustering analysis using the FPKM values of differentially expressed genes under different experimental conditions as indicators of the expression levels. The different-colored areas represent different clustering information, and the genes within the same group have similar expression patterns and may have similar functions or participate in the same biological processes.

### Effect of lncRNAs on the Up- and Downregulation of Differentially Expressed Genes

LncRNAs have different modes of action on target genes, resulting in different regulatory effects on target genes (including positive and negative). Combined with the specific up- and downregulation information of differential lncRNAs between samples, the specific up- and downregulation information of target genes corresponding to differentially expressed lncRNAs was analyzed by the following rules. The lncRNA was upregulated when there was positive action on the corresponding target gene, and the target gene was ultimately marked as upregulated; the lncRNA was upregulated when there was negative action on the corresponding target gene, and the target gene was ultimately marked as downregulated; the lncRNA was downregulated when there was positive action on the corresponding target gene, and the lncRNA was downregulated when there was negative action on the corresponding target gene, and the target gene is ultimately marked as upregulated.

### GO Enrichment Analysis of Differentially Expressed Genes

All differentially expressed genes were first mapped to each term of the Gene Ontology database, the number of genes per term was calculated, and then a hypergeometric test was applied to identify GO entries that were significantly enriched in differentially expressed genes compared to the whole genomic background. The calculated p values were corrected by the Bonferroni method with a threshold of corrected p value ≤ 0.05, and GO terms that met this condition were defined as GO terms that were significantly enriched in differentially expressed genes. The GO functionally significant enrichment analysis enabled the identification of the major biological functions exercised by differentially expressed genes.

### KEGG Enrichment Analysis of Differentially Expressed Genes

In organisms, different genes coordinate their biological functions with each other, and significant pathway enrichment can identify the most important biochemical metabolic pathways and signal transduction pathways in which differentially expressed genes are involved. KEGG is the main public database for pathway information (Mortazavi et al., 2008). Pathway significant enrichment analysis uses KEGG pathways as units and applies hypergeometric tests that identify pathways that are significantly enriched in differentially expressed genes compared to the whole genomic background.

### Transcriptome-Wide Association Analysis

miRNAs have the ability to inhibit the transcription and translation of target mRNAs or to shear target mRNAs and promote their degradation. Both lncRNAs and circRNAs can act as ceRNAs to bind to microRNAs to regulate mRNA expression. lncRNAs can transcriptionally regulate mRNA expression in cis or in trans and can also act as miRNA sponges to competitively bind miRNAs in posttranscriptional regulation, inhibiting the miRNA regulation of target genes. The source genes of circRNAs can also be transcribed to form linear mRNAs, and changes in circRNA expression levels may affect changes in the expression levels of the mRNAs of their source genes. Based on ceRNA theory, lncRNA-mRNAs that share the same miRNA-binding site were searched for, and lncRNA-miRNA-mRNA combinations were screened. Based on lncRNA-miRNA-mRNA combinations and circRNA-miRNA-mRNA combinations, taking miRNAs as intersection nodes, potential miRNAs that are coregulated by both lncRNAs and circRNAs can be identified. For lncRNAs that regulate mRNAs, including epigenetic regulation, transcriptional regulation and posttranscriptional regulation, we analyzed Hi-C and ATAC-seq data.

### Candidate Target Gene Analysis

Candidate target genes obtained by taking intersections of differentially expressed mRNAs as well as target genes that may be regulated by ceRNAs in the results of the association analysis were subjected to gene clustering, GO enrichment, and KEGG enrichment analyses.

### Statistical Analysis

All results are expressed as the mean ± standard deviation (SD) of three independent experiments. Statistical analysis was performed using the t test. All statistical tests were two-tailed, and P<0.05 was considered significant.

## Results

### LncRNAs Expression Analysis

LncRNAs and their target genes prediction step (**Supplementary Figure 1A**), resulting in structure and sequence information statistics for all lncRNAs, for both known and unknown lncRNA statisticswe can see that unknown lncRNAs account for approximately 80% of the total lncRNAs, while the known lncRNAs account for only 20% of the total lncRNAs (**Supplementary Figure 1B**). The distribution of length of the lncRNA sequences is primarily greater than 2000 (**Supplementary Figure 1C**). Approximately 65% of the lncRNAs contained 2 exons and the rest contained 3 or more exons (**Supplementary Figure 1D**). The results of the lncRNA species distribution show that intergenic lncRNAs account for approximately 46% and antisense lncRNAs account for approximately 44%, but intronic lncRNAs account for only approximately 10% (**Supplementary Figure 1E**). A total of 3543 cis-acting model target genes (**Supplementary Table 1**) and 2682 trans-acting model target genes were found (**Supplementary Table 2**). By comparing the lncRNA gene expression levels under different experimental conditions, the FPKM distribution map (**Supplementary Figure 1F**) and the box map (**Supplementary Figure 1G**) were obtained.

### Expression Analysis of Differential LncRNAs

**Figure 1:**
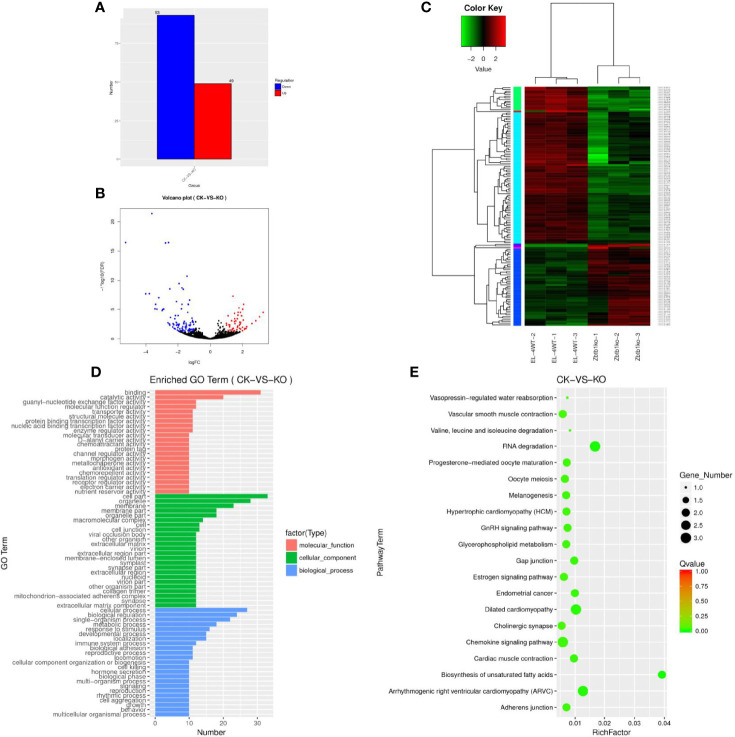
**(A)** Sample difference comparing the upregulation and downregulation of lncRNA gene expression. **(B)** Volcano map of differential lncRNA genes. Red dots indicate significantly upregulated genes, and blue dots indicate downregulated expression. The abscissa represents multiple changes in gene expression in different samples; the ordinate represents statistically significant changes in gene expression. **(C)** Differential lncRNA gene cluster map clustered by log10 (FPKM+1) values; red indicates highly expressed genes, and green represents genes with low expression. As the color shifts from red to blue, a higher gene expression is indicated. **(D)** The differential lncRNA target gene GO enrichment histogram and the ordinate enrichment GO term. The abscissa is the number of differential genes in the term. Different colors are used to distinguish biological processes, cellular components and molecular functions. **(E)** KEGG enrichment and scatter map of differential lncRNA target genes. The vertical axis represents the pathway name, the horizontal axis indicates the size of the rich factor, the dot indicates the number of differentially expressed genes in this pathway, and the color of the dot corresponds to different Q value ranges.

The statistics of the upregulated and downregulated expression of the lncRNA genes showed that 49 genes were upregulated and 93 genes were downregulated in the KO group compared with the CK group (**Supplementary Table 3**). A map of differentially expressed genes (**Figure 1A**) and a volcano map of differentially expressed genes (**Figure 1B**) were drawn. Through a cluster analysis of the differentially expressed lncRNA genes indicates that the upregulated and downregulated genes may have similar functions or participate in the same biological process (**Figure 1C**).

### GO Enrichment and KEGG Enrichment of LncRNA Target Genes

A GO analysis of the lncRNA target genes revealed 69 enriched GO terms (**Figure 1D**), the cell biology processes that were significantly enriched were biological regulation, cellular processes, and single-organism processes. These results suggest that lncRNAs may play an important role in the catalysis of cell metabolism, cell composition and biological regulation. KEGG enrichment analysis of the differentially expressed lncRNA target genes showed that 30166 lncRNA target genes (**Supplementary Table 4**) were enriched in 38 pathways (**Supplementary Table 5**). We selected the 20 pathways with the most significant enrichment for display in the KEGG enrichment scatter map (**Figure 1E**). It can be seen that for biological systems, specifically in types of diseases, metabolism and other related signal pathways, the two pathways of RNA degradation and biosynthesis of unsaturated fatty acids were significantly enriched. The correct handling, quality control and updating of RNA degradation and cellular RNA molecules are very important for the expression of genetic information.

### Expression Analysis of Differential mRNAs

**Figure 2:**
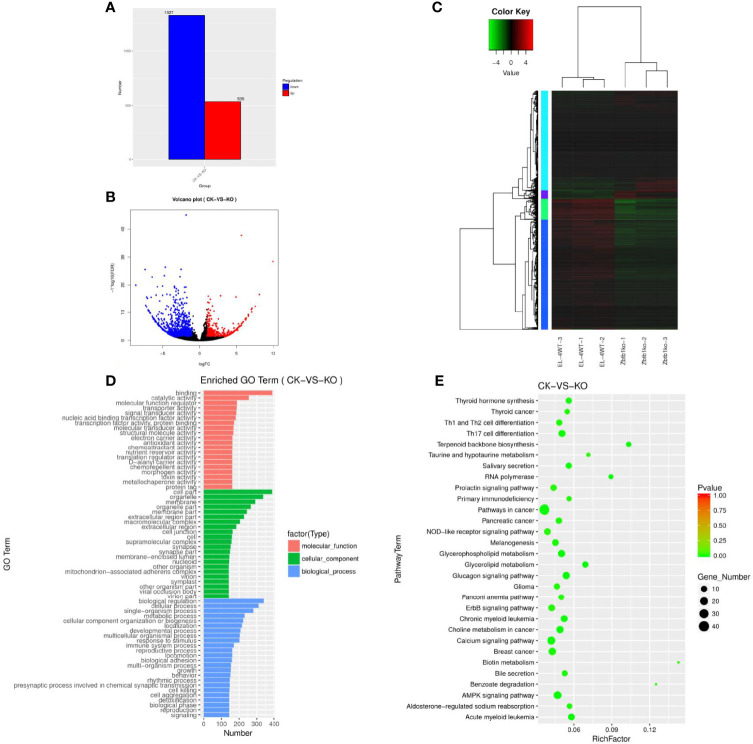
**(A)** Sample difference comparing the upregulation and downregulation of mRNA gene expression. **(B)** Volcano map of differentially expressed mRNAs. Red dots indicate significantly upregulated genes, and blue dots indicate downregulated genes. The abscissa represents multiple changes in gene expression in different samples; the ordinate represents statistically significant differences in gene expression. **(C)** Differential mRNA gene cluster map clustered by log10 (FPKM+1) values; red indicates highly expressed genes, and green represents genes with low expression. As the color shifts from red to blue, higher gene expression is indicated. **(D)** The differential mRNA target gene GO enrichment histogram and the ordinate enrichment GO term. The abscissa is the number of differential genes in the term. Different colors are used to distinguish biological processes, cellular components and molecular functions. **(E)** Differential gene KEGG enrichment and scatter map. The vertical axis represents the pathway name, the horizontal axis indicates the size of the rich factor, the dot indicates the number of differentially expressed genes in this pathway, and the color of the dot corresponds to different P value ranges.

The mRNA gene expression levels under different experimental conditions were compared by an FPKM distribution map (**Supplementary Figure 1H**) and a box diagram (**Supplementary Figure 1I**). The structure of mRNA was compared with that of lncRNA, the length of mRNA and lncRNA was observed (**Supplementary Figure 1J**), and the number of exons was compared (**Supplementary Figure 1K**). A total of 103639 mRNAs (**Supplementary Table 6**) were enriched, among which 1862 genes (**Supplementary Table 7**) were significantly differentially expressed. A gene map (**Figure 2A**) and a volcano map (**Figure 2B**) of the differentially expressed genes were drawn, and the gene cluster analysis results of the differentially expressed mRNA showed that upregulated and downregulated genes may have similar functions or participate in the same biological process (**Figure 2C**).

### GO Enrichment and KEGG Enrichment of mRNAs

The GO enrichment histogram of the mRNAs of the differentially expressed genes suggests that mRNAs may play an important role in the catalysis of cell metabolism, cell composition and biological regulation (**Figure 2D**). A total of 31676 differentially expressed mRNAs showed KEGG enrichment (**Supplementary Table 8**), and 30 pathways with significant enrichment were selected to draw a KEGG enrichment scatter map (**Figure 2E**), which showed that these genes were significantly enriched in biological systems, specific disease, environmental information processing, cancer and other signaling pathways and were highly enriched in terpenoid backbone biosynthesis, RNA polymerase, biotin metabolism, benzoate degradation and other pathways.

### Results of the Association Analysis

**Figure 3:**
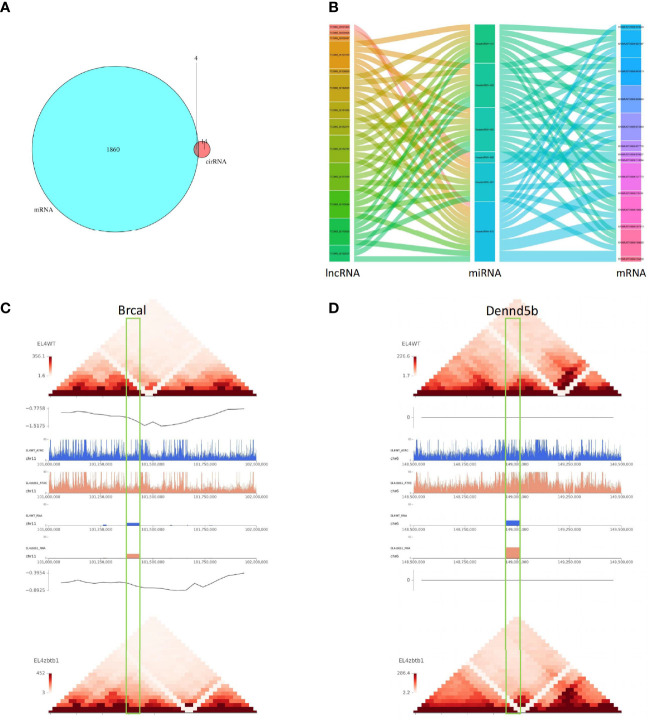
**(A)** The intersection of differentially circRNA-derived genes and differentially expressed mRNAs. **(B)** The sankey diagram used for data association analysis, from left to right data categories are lncRNA, miRNA, mRNA, different color blocks in each column are different components in the corresponding category, and the connecting lines between components indicate the association between groups. **(C)** Comparison of pictures of the region of the Brca1 gene on the chromosome between the CK and KO groups. 0.5M upstream and downstream of selected genes for display. Under the folder are multi-omics joint display images of two genes. The first layer is a heat map of the regional interactions of EL4WT. The second layer is the visualization of the insulation score of EL4WT. Layers 3-6 show the signal values of ATAC and RNA for the two samples in the corresponding regions. Layer seven is the visualization of the insulation score of EL4zbtb1. Layer 8 is the regional interaction heat map of EL4zbtb1. **(D)** Comparison of pictures of the region of the Dennd5b gene on the chromosome between the CK and KO groups.

Twenty-four differentially expressed microRNAs had possible regulatory relationships with 1096 differentially expressed mRNAs (**Supplementary Table 9**). Differentially expressed lncRNAs had possible regulatory relationships with 120 target genes (**Supplementary Table 10**); 72 lncRNAs could upregulate 76 of their target genes, and 39 lncRNAs could downregulate 44 of their target genes. The intersection of differential circRNA source genes and differentially expressed mRNAs was taken, and the differential expression of circRNAs between samples was found to reflect changes in the expression levels of the corresponding mRNAs of the source genes (**Figure 3A**). The results of the lncRNA-miRNA-mRNA association analysis are shown in **Figure 3B** (**Supplementary Table 11**).

### Analysis of mRNAs Directly Regulated by lncRNAs

**Figure 4:**
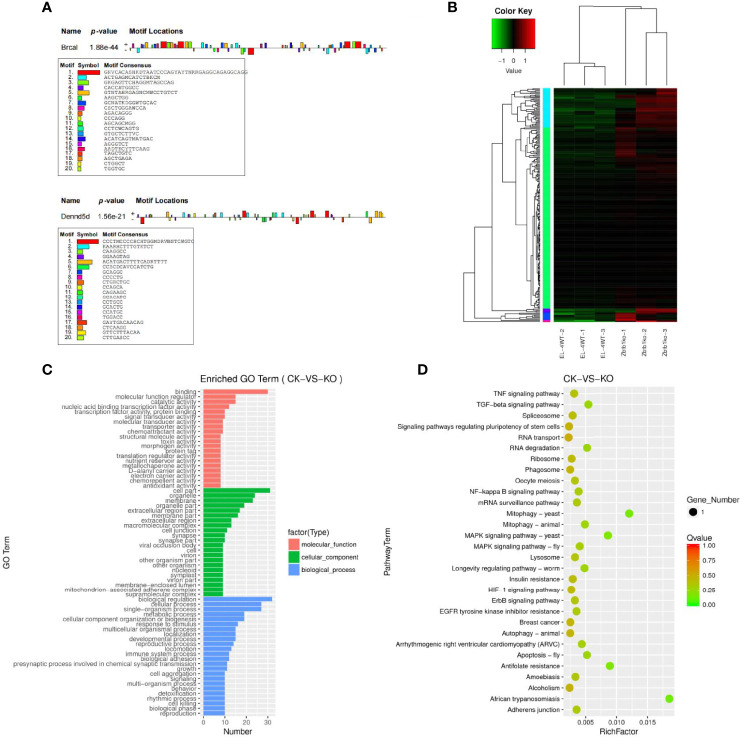
**(A)** Motif statistical analysis of the promoter regions of the Brca1 and Dennd5b genes. **(B)** Candidate target genes cluster map clustered by log10 (FPKM+1) values; red indicates highly expressed genes, and green represents genes with low expression. As the color shifts from red to blue, higher gene expression is indicated. **(C)** Candidate target genes GO enrichment histogram and the ordinate enrichment GO term. The abscissa is the number of differential genes in the term. Different colors are used to distinguish biological processes, cellular components and molecular functions. **(D)** Candidate target genes KEGG enrichment and scatter map. The vertical axis represents the pathway name, the horizontal axis indicates the size of the rich factor, the dot indicates the number of differentially expressed genes in this pathway, and the color of the dot corresponds to different P value ranges.

The predicted results of target gene mRNAs of differentially expressed lncRNAs and the actual expression of mRNAs were taken as intersections, and regulatory interference between circRNA, microRNA and mRNA was excluded. Twelve lncRNAs were eventually identified that may have a role in regulating the expression of mRNAs (**Supplementary Table 12**), of which six lncRNAs can upregulate the expression of target genes through cis activity and six lncRNAs can downregulate the expression of target genes through trans activity. Unfortunately, the differential expression of genes downregulated by lncRNA action was not significant, and the significantly differentially expressed genes upregulated by lncRNA action included Brca1 and Dennd5b. By joint Hi-C and ATAC-seq correlation data analysis, we showed that the spatial structure and chromatin opening of the gene location expressing Brcal did not differ significantly when comparing the CK and KO groups (**Figure 3C**) and thus may be posttranscriptionally regulated by lncRNAs, while the TAD boundary at the location of the Dennd5b gene is altered and chromatin opening is enhanced (**Figure 3D**), reminiscent of epigenetic regulation by lncRNAs. We also obtained the binding sites (motifs) of transcription factors and other DNA sequences in differential open regions, identified and annotated motifs in open regions specific to the groups, and found that although there were more motifs in the promoter regions of the Brcal and Dennd5d genes (**Figure 4A** and showing the top 20), the open regions in the control specific motifs were not found in the results (**Supplementary Figure 2**). Most of the lncRNA-regulated mRNAs shown in our results are currently involved in only some GO pathways, but their specific functions and the roles they play in the pathway remain to be demonstrated.

### Candidate Target Gene Analysis

The results of target genes predicted to be regulated by ceRNAs and mRNAs that actually underwent significant differential expression were intersected to obtain 228 candidate target genes (**Supplementary Table 13**), subject to clustering analysis of the screened candidate target genes (**Figure 4B**), and the results of the GO enrichment analysis for the candidate target genes with significant differential expression were not much different from the GO enrichment results for differentially expressed mRNAs (**Figure 4C**). KEGG enrichment analysis was performed for the differentially expressed candidate target genes, and the results revealed that the candidate target genes were not enriched in pathways and mostly functioned as single genes. Significantly enriched pathways were pathways such as African trypanosomiasis and mitophagy-yeast (**Figure 4D**).

## Discussion

Zbtb1 not only plays an important role in lymphocyte development but has also been associated with cancer and DNA damage repair (Siggs et al., 2012; Kim et al., 2014). Our previous studies identified differential expression of Zbtb1 in pancreatic cancer (Cheng et al., 2021a); a reciprocal inhibitory role between IL-7Rα signalling (Cao et al., 2018); protection of genomic integrity and prevention of p53-mediated apoptosis in proliferating lymphoid progenitor cells (Cao et al., 2016); and differential expression of microRNA and circRNA in Zbtb1 knockdown monoclonal EL4 cells (Wang et al., 2021). Recent studies have shown that the ZBTB gene family is a key regulator of T cell development, differentiation and effector functions (Cheng et al., 2021b). As people continue to study the ZBTB gene family, an increasing number of mechanisms of action and regulatory functions are being revealed.

The understanding of lncRNAs is still in its infancy. lncRNAs were initially considered “noise” of genomic transcription, a byproduct of RNA polymerase II transcription, and had no biological function (Yang and Meng, 2019). However, it was found that some long noncoding RNAs are only expressed at specific stages of eukaryotic development and are closely related to the occurrence of human diseases, including cancer and degenerative neurological diseases, which are serious human health hazards manifested by certain abnormal sequences and spatial structures of long noncoding RNAs, abnormal expression levels, and abnormal interactions with binding proteins (Li et al., 2021; Ma et al., 2021). For lncRNAs, recent studies have shown that lncRNA function depends on their binding to various regulatory elements (Zhang G. et al., 2019).

The differentially expressed genes Brca1 and Dennd5d in this study may be regulated by lncRNA, and Brca1 has multiple functions, including double-stranded DNA break repair, involvement in genomic surveillance, transcription-coupled DNA repair, transcriptional regulation, chromatin remodeling, and ubiquitin ligation and cell cycle checkpoint blockade. In cells, loss of Brca1 function results in spontaneous chromosome breaks and susceptibility to DNA damage (Wang et al., 2021). As a gene directly related to hereditary breast cancer, the possibility of a regulatory relationship between Brca1 and lncRNA is high and may be a potential biomarker for breast cancer and a therapeutic target (Wang et al., 2019). Dennd5b was identified as a prognostic biomarker in colorectal cancer (Zhang Z. et al., 2019) and is involved in the regulation of intestinal triglyceride absorption and body weight (Gordon et al., 2019). Our studies of chromosomal structural changes and open region changes in the Brca1 and Dennd5d genes revealed that Brca1 may be posttranscriptionally regulated by lncRNAs, while Dennd5b is more likely epigenetically regulated by lncRNAs.

We analyzed the whole transcriptome (transcriptome) products of Zbtb1 knockdown EL4 cell lines by combining the differentially expressed mRNAs, lncRNAs, circRNAs, and small RNAs in KO and CK group samples and finally constructed a lncRNA-mediated ceRNA network.

In future studies, it will be necessary to identify the specific regulatory mechanisms of lncRNAs for these two target genes (Brca1 and Dennd5d), especially Brca1, which might identify potential biomarkers and therapeutic targets for breast cancer.

## Data Availability Statement

The datasets presented in this study can be found in online repositories. The names of the repository/repositories and accession number(s) can be found in the article/**Supplementary Material**.

## Author Contributions

XC, JW, and CW conceived and designed research. JW and XL conducted experiments. FL and YL analyzed data. QH and MC wrote the manuscript. YS, CS, and YZ contributed to the work. All authors contributed to the article and approved the submitted version.

## Funding

This work was supported by the National Natural Science Foundation of China (81760287,31941018), the Science and Technology Development Program of Jilin Province (20200402041NC, YDZJ202102CXJD029), Science and Technology Project of the Education Department of Jilin Province during the 13th Five-year Plan (JJKH20200360KJ), China Agriculture Research System of MOF and MARA.

## Conflict of Interest

The authors declare that the research was conducted in the absence of any commercial or financial relationships that could be construed as a potential conflict of interest.

## Publisher’s Note

All claims expressed in this article are solely those of the authors and do not necessarily represent those of their affiliated organizations, or those of the publisher, the editors and the reviewers. Any product that may be evaluated in this article, or claim that may be made by its manufacturer, is not guaranteed or endorsed by the publisher.

## Supplementary Material

Supplementary Figure 1
**(A)** Flow chart for lncRNA identification and prediction. **(B)** The known and unknown lncRNA information contained in the sample. **(C)** The statistical distribution of the lengths of lncRNAs contained in the sample. **(D)** The statistical distribution of the number of exons in the lncRNAs contained in the sample. **(E)** The distribution of different lncRNA species in the sample. **(F)** The lncRNA gene expression level was compared with the FPKM distribution map under different experimental conditions. The abscissa was the log10 (FPKM), and the ordinate and gene density are shown. **(G)** Under different experimental conditions, the lncRNA gene expression level was compared with the FPKM box map, the abscissa is the sample name, and the ordinate is the box map of each region of log10 (FPKM) with the five-number summary (from top to bottom are the maximum, the upper quartile, the median, the lower quartile and the minimum). **(H)** Under different experimental conditions, the mRNA gene expression level was compared to the FPKM distribution map, and the abscissa is the log10 (FPKM), and the ordinate is the gene density. **(I)** Under different experimental conditions, the mRNA gene expression level was compared to the FPKM box map, the abscissa is the sample name, and the ordinate is the box map of each region of log10 (FPKM) with the five-number summary (from top to bottom are the maximum, the upper quartile, the median, the lower quartile and the minimum). **(J)** The statistical distribution of the length of transcripts of different types contained in all samples, where green indicates mRNA, red indicates lncRNA, and blue indicates novel RNA. **(K)** The statistical distribution of the number of exons contained in different types of transcripts in all samples, where green indicates mRNA, red indicates lncRNA, and blue indicates novel RNA.

Supplementary Figure 2 Summary plot of motifs specific to differentially open regions of the CK group

Supplementary Table 1 (1) LncRNA_geneID: LncRNA gene ID; (2) LncRNA_ID: LncRNA ID; (3) TargetGeneID: target gene ID; (4) the number of overlapping bases between NumberBasesMapped and the target gene promoter; (5) the regulation of effects on the target gene.

Supplementary Table 2 (1) ID of LncName: LncRNA; (2) Transcript name of TargetTranscriptID: action; (3) Regulation of effects on target genes.

Supplementary Table 3 Statistics for the significant differential expression of lncRNA genes that are upregulated and downregulated.

Supplementary Table 4 Specific effects of differentially expressed lncRNAs on target gene statistics.

Supplementary Table 5 KEGG enrichment list of differentially expressed lncRNA target genes (1) PathwayID: pathway ID; (2) Pathway: pathway name; (3) DEGs with pathway annotation: the number of genes in the differential analysis comparison group; (4) All genes with pathway annotation: the number of genes in the genetic background; (5) P value: this pathway enriches the P value; (6) Q value: this pathway enriches the Q value; (7) Genelist: this pathway enriches the list of differential genes; (8) KOlist: this pathway enriches the KO list of differential genes.

Supplementary Table 6 Statistics for the lncRNA target gene enrichment pathways.

Supplementary Table 7 Enrichment in the statistics of total differentially expressed mRNA genes: (1) the average normalization of the number of IDs; (2) baseMean: fragments of Id: genes and the average value under two conditions; (3) log2FoldChange: takes log; (4) lfcSE: to estimate the standard error of log2FoldChange based on 2; (5) stat: the Wald test; (6) significant statistical differences in the p value; (7) padj: FDR correction P value.

Supplementary Table 8 31676 differentially expressed mRNAs showed KEGG enrichment.

Supplementary Table 9 Differentially expressed miRNA and its differentially expressed target mRNA statistics: (1) the up-down relationship of ID; (2) miRNA_up/down: miRNA of miRNA_ID: miRNA; (3) the up-down relationship of ID; (4) mRNA_up/down: mRNA of mRNA_ID: miRNA target mRNA.

Supplementary Table 10 lncRNAs transcribed the expression of mRNA (up- and downregulated by 1) lncRNA: lncRNA ID; (2) Target: mRNA ID.

Supplementary Table 11 The results of lncRNA-miRNA-mRNA association analysis.

Supplementary Table 12 The 12 lncNRAs obtained by screening may play a regulatory role in regulating the expression of mrna.

Supplementary Table 13 Statistical table of 228 candidate target genes.
