# Supplementary figures and images for: Analysis of lncRNAs and mRNA Expression in the ZBTB1 Knockout Monoclonal EL4 Cell Line and Combined Analysis With miRNAs and circRNAs
Source: Front Cell Infect Microbiol. 2021 Dec 9;11:806290. doi: 10.3389/fcimb.2021.806290 (PMC8695857; doi:10.3389/fcimb.2021.806290)

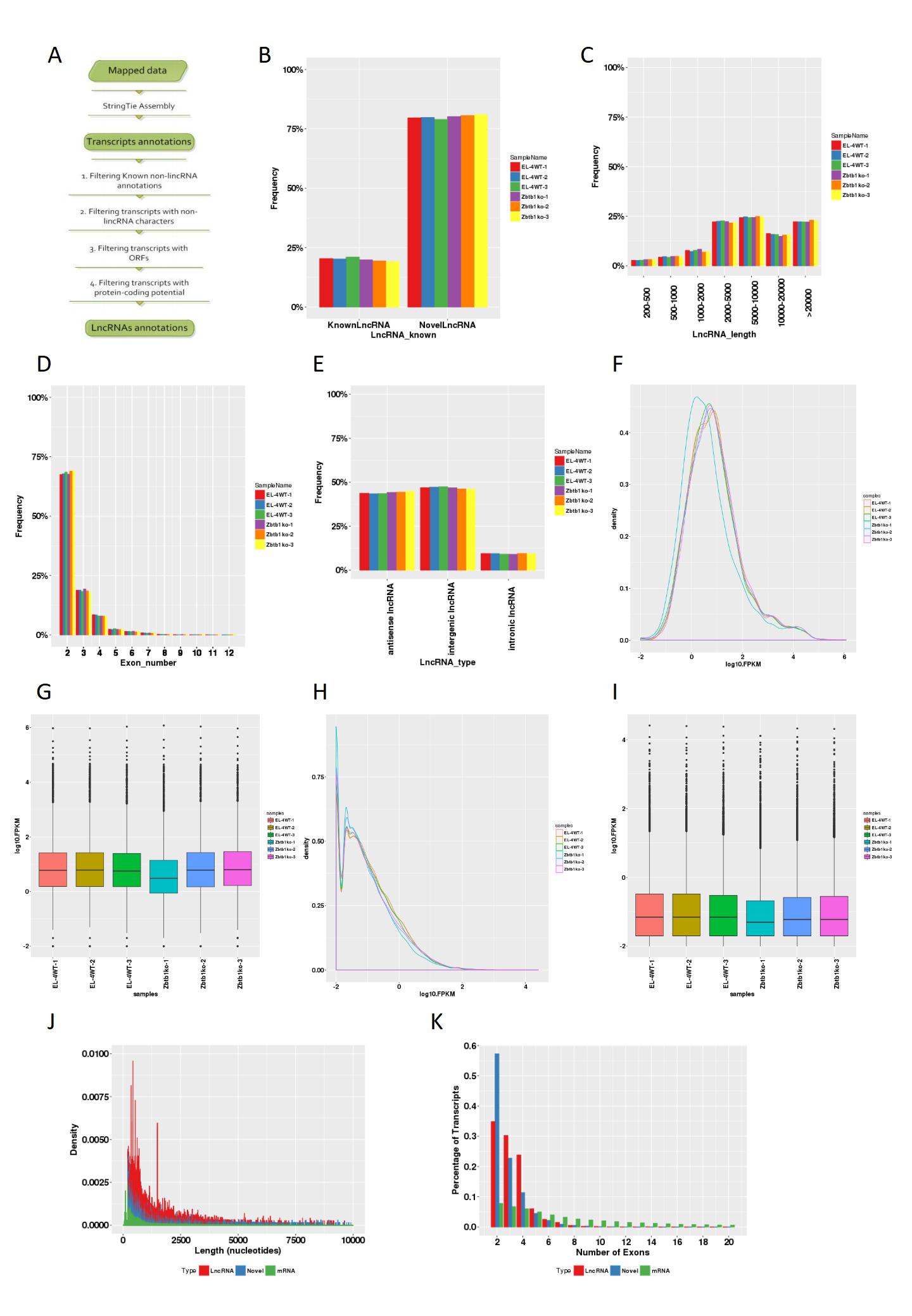

Supplement: Supplementary Figure 1 — (A) Flow chart for lncRNA identification and prediction. (B) The known and unknown lncRNA information contained in the sample. (C) The statistical distribution of the lengths of lncRNAs contained in the sample. (D) The statistical distribution of the number of exons in the lncRNAs contained in the sample. (E) The distribution of different lncRNA species in the sample. (F) The lncRNA gene expression level was compared with the FPKM distribution map under different experimental conditions. The abscissa was the log10 (FPKM), and the ordinate and gene density are shown. (G) Under different experimental conditions, the lncRNA gene expression level was compared with the FPKM box map, the abscissa is the sample name, and the ordinate is the box map of each region of log10 (FPKM) with the five-number summary (from top to bottom are the maximum, the upper quartile, the median, the lower quartile and the minimum). (H) Under different experimental conditions, the mRNA gene expression level was compared to the FPKM distribution map, and the abscissa is the log10 (FPKM), and the ordinate is the gene density. (I) Under different experimental conditions, the mRNA gene expression level was compared to the FPKM box map, the abscissa is the sample name, and the ordinate is the box map of each region of log10 (FPKM) with the five-number summary (from top to bottom are the maximum, the upper quartile, the median, the lower quartile and the minimum). (J) The statistical distribution of the length of transcripts of different types contained in all samples, where green indicates mRNA, red indicates lncRNA, and blue indicates novel RNA. (K) The statistical distribution of the number of exons contained in different types of transcripts in all samples, where green indicates mRNA, red indicates lncRNA, and blue indicates novel RNA. [file Image_1.jpeg]

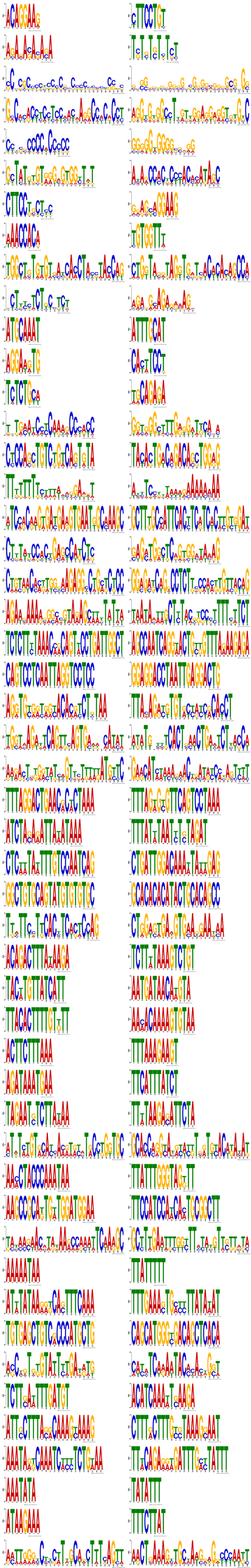

Supplement: Supplementary Figure 2 — Summary plot of motifs specific to differentially open regions of the CK group [file Image_2.png]
